# Crystal diffraction prediction and partiality estimation using Gaussian basis functions

**DOI:** 10.1107/S2053273323000682

**Published:** 2023-02-17

**Authors:** Wolfgang Brehm, Thomas White, Henry N. Chapman

**Affiliations:** a Center for Free-Electron Laser Science CFEL, Deutsches Elektronen-Synchrotron DESY, Notkestrasse 85, 22607 Hamburg, Germany; b Department of Physics, Universität Hamburg, Luruper Chaussee 149, 22761 Hamburg, Germany; c The Hamburg Centre for Ultrafast Imaging, Luruper Chaussee 149, 22761 Hamburg, Germany; University of Patras, Greece

**Keywords:** partiality estimation, diffraction prediction, merging, serial snapshot crystallography

## Abstract

The recent diversification of macromolecular crystallographic experiments including the use of pink beams, convergent electron diffraction and serial snapshot crystallography has shown the limitations of using the Laue equations for diffraction prediction. This article gives a computationally efficient way of calculating approximate crystal diffraction patterns given varying distributions of the incoming beam, crystal shapes and other potentially hidden parameters. This approach models each pixel of a diffraction pattern and improves data processing of integrated peak intensities by enabling the correction of partially recorded reflections. The fundamental idea is to express the distributions as weighted sums of Gaussian functions. The approach is demonstrated on serial femtosecond crystallography data sets, showing a significant decrease in the required number of patterns to refine a structure to a given error.

## Abstract 2

Reflection position, size and shape prediction and partiality estimation of crystal diffraction by integrating using a Gaussian basis are described.

## 1. Introduction

Macromolecular crystallography is most commonly performed using a monochromatic X-ray or electron source and with at most a few crystals. In conventional rotation measurements each crystal is rotated, exposing it to the beam over a range of about 180°, integrating the diffraction over small angular wedges. Under those circumstances the Laue equations have been sufficient approximations for the diffraction condition. They stipulate that the differences 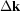 between the wavevector of the diffracted beam 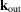 and the wavevector of the incident beam 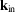 are integer linear combinations of the reciprocal unit-cell vectors 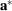 , 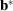 and 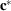 : 

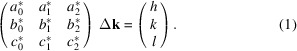

Given the unit-cell parameters, initial crystal orientation and experimental geometry, the equation can be rearranged to give the crystal orientation and the point on the detector where a given reflection can be observed most intensely. Conversely, for a random orientation of the crystal, the probability of any reflection (except the direct beam) being in its optimal diffraction condition is zero because the integer indices on the right side of equation (1) are an infinitesimal subset of the attainable rational vectors on the left side. Experimentally however, there is a neighbourhood close to the ideal diffraction condition where diffraction can be observed at reduced intensity even though the Laue equations are not satisfied. Not knowing which reflections will be observable for a given orientation, and how intensely, is known as the partiality problem. Several definitions of partiality are conceivable. In the following the partiality of an observation will be the ratio between the measured intensity and the maximally attainable intensity for a given crystal and beam but changing the orientation of the crystal. This paper introduces a way to estimate that neighbourhood and the reduction in intensity, thereby addressing the partiality problem computationally.

Exposing the crystal to the radiation during rotation and recording images over small angular wedges solves this problem too, which is why the rotation method was adopted in the first place. The rotation ensures that almost all reflections within the observable resolution range of the diffractometer will reach their optimum at some point during the rotation and can be fully recorded. The process of calculating any or all aspects of diffraction patterns (peak position, shape, intensity or full diffraction patterns), given unit-cell parameters and experimental geometry, is called ‘prediction’ in the context of macromolecular crystallography data processing. For monochromatic rotational crystallography the deviations between measured and predicted peak positions are usually small, except for reflections whose reflection condition is not affected significantly by the rotation. (Those few measurements are typically discarded.) The rotation of the crystal during the exposure about a known axis and with a known angular increment acts as a strong constraint for parameter estimation during the processing of rotational crystallographic data. Using this information, the intensity of a reflection can be integrated and corrected to yield the corresponding squared structure-factor amplitude.

In the last decades in macromolecular crystallography, methods have been employed, which, for various reasons, deviate from the rotational crystallography setup in significant ways. The most notable among these methods is serial crystallography, where crystals are recorded once each and consequently many crystals are needed for a complete data set (Schlichting, 2015 ▸; Spence, 2017 ▸). An important subclass is serial snapshot crystallography, where the crystals are illuminated without rotation. Without the rotation it becomes indispensable to consider not just the ideal diffraction condition, but the partial intensity that can be observed when close enough to the ideal diffraction condition.

We know there is a steep fall-off of intensity with deviation from the exact condition in a monochromatic experiment with well ordered crystals. This steep fall-off makes it easy to define a small range that contains almost all observations of the same structure factor and hardly any observations of anything else, even without knowing the shape of the fall-off. Computing the average of these observations with unknown partiality is called Monte Carlo integration in the context of serial crystallography. It has been used to work around the problem of unknown partial intensities with great success (Kirian *et al.*, 2011 ▸). However, for the Monte Carlo integration to converge to an average with a small standard deviation, each reflection needs to be measured multiple times. This approach assumes that the partialities follow the same distribution, with finite first and second moments, for all reflections of a given resolution shell. From this assumption it follows that the average converges to a value proportional to the non-partial intensity, that is the structure-factor amplitudes squared. Assuming polarization correction has been applied before averaging, no additional correction factors are needed, unless the inclusion criterion varies or fails to capture a significant portion of the intensity, and in fact no Lorentz factor is applied in practice.

The development of new methods has not stopped there, however. Serial snapshot crystallography has since been carried out with polychromatic, or so-called pink beam, sources (Meents *et al.*, 2017 ▸), electron beams (Bücker *et al.*, 2020 ▸) and mosaic crystals. More exotic experiments are surely already planned. In these more general cases the Laue equations are not sufficient, because inaccurate predictions of the peak positions and elongated peak shapes cannot necessarily be overcome by just measuring several times more data to make use of Monte Carlo integration. The Laue equations assume point-like peak shapes. In monochromatic experiments the peaks are narrow and compact, so small integration radii or boxes are typically employed, and the Laue equations are sufficient. But when two or more different and equally significant distributions are at play, elongated peak shapes can be observed.

**Figure 1:**
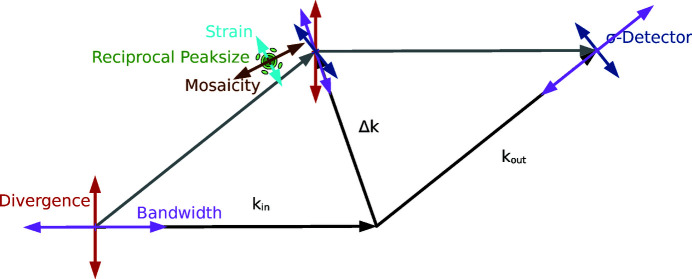
The geometric construction visualizing the construction of the covariance matrices of the distributions of diffractive power in reciprocal space and the volume probed by an incident beam. The arrows indicate the components, akin to error bars, that the different distributions contribute to the covariance matrix in a 2D cut. The same contributions have a different effect on 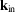 , 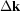 and 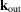 , and where they have an effect they are indicated with the same colour as where they were introduced. The distribution of wavelengths in the incident beam leads to a distribution of lengths of 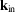 ; the standard deviation is drawn with purple arrows. The distribution of incident-beam directions leads to different starting points of 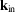 in the Ewald construction; its standard deviation is drawn in red. The scattering power of the crystal is smeared rotationally by mosaicity, drawn with brown arrows, and smeared radially by (a simplified) strain, drawn in cyan. The reciprocal peak shape as depicted in light green is a stylized shape transform, which too will be approximated as a Gaussian. To smooth the prediction over a range of output directions in order to simulate the detector point spread function and facilitate efficient sampling of the signal, a distribution of diffraction directions can be introduced, the standard deviation of which is drawn in dark blue.

Fig. 1 ▸ depicts the distributions that are assumed to be relevant and their effect on the diffraction geometry. In polychromatic experiments the distribution of wavelengths, the width of which is called bandwidth, together with a distribution in crystal orientation, called mosaicity, can lead to elongated peak shapes. The other relevant distributions affecting the diffraction are the size and shape of the crystal (reciprocal peak size), convergence (or divergence) of the beam, and different strain throughout the crystal (which is a variation of unit-cell parameters throughout the crystal volume). Once there is more than one relevant distribution, the exact location of the peak on the detector can no longer be determined solely by rearranging the Laue equations. This paper shows how to model diffraction efficiently in a way that generalizes to these different conditions, by first introducing an approximation for calculating full diffraction patterns and then deriving from that peak locations, shapes and estimates for their total intensity. Two applications of this model are presented in Sections 4 and 5. In Section 4 diffraction patterns are approximated in full detail, pixel by pixel. Optimizing the free parameters of the model to fit the diffraction pattern in each pixel should determine the structure-factor amplitudes in the most efficient way, in terms of diffraction data needed and achievable precision. This may provide an insight into the relatively small heterogeneity between samples, which has proven to be elusive in the presence of large data processing artifacts and measurement errors. The second application is more conventional. In Section 5 an expression for the partial intensity of a reflection in a ‘still’ diffraction pattern (that is, one recorded from a static crystal without rotation) is derived and used to correct serial crystallographic data sets, improving the convergence rate of merging the intensity data to determine the structure factors.

## 2. Previous approaches

The earliest approaches to dealing with partially recorded reflections relied upon the redundancy afforded by rotation experiments, which makes them inapplicable in serial crystallography. Under those conditions the partiality as a function of the crystal rotation can be reconstructed as a smooth function, because it is overdetermined by the diffraction data. Using the reconstructed profile, the partially observed reflections can be corrected (Diamond, 1969 ▸; Grant & Gabe, 1978 ▸; Winkler *et al.*, 1979 ▸).

An early approach in dealing with partial reflections that can be applied to single diffraction patterns (Rossmann *et al.*, 1979 ▸) assumed reciprocal peaks to be spheres. While the diffraction process is modelled similarly to the earlier approaches with the intersection of these small spheres with the Ewald sphere, here the rocking curve is determined entirely by the intersection of the Ewald sphere with the reciprocal-lattice spheres. The reduction allows us to use this model even for single diffraction patterns. Greenhough and Helliwell continued this approach and have generalized it to ellipsoidal shapes (Greenhough & Helliwell, 1982*a* ▸,*b* ▸; Greenhough *et al.*, 1983 ▸). Andrews *et al.* (1987) ▸ showed that this approach can even be applied to Laue diffraction (with very high polychromaticity). The model of Rossmann *et al.* was generalized by Ginn *et al.* (2015) ▸ with a super-Gaussian distribution of Ewald spheres given by the distribution of wavelengths and incidence angles, requiring a numerical integration that is efficiently implemented in *CrystFEL* (White *et al.*, 2016 ▸) as the partiality model xsphere. This model has 11 free parameters per crystal in total: nine for the unavoidable unit-cell matrix and one each for the mosaicity radius and the profile radius.

Holton *et al.* (2014) ▸ modelled the most relevant contributions, save the crystal shape transform, based on the principles laid out by Greenhough & Helliwell (1983) ▸ and Winkler *et al.* (1979) ▸ (modelling mosaicity with the intersection of a disc with the Ewald sphere). They also used Gaussian basis functions, but instead of analytical integration of the different distributions, they computed numerical integrals to combine different effects with automatic sampling. No attempt to match measured diffraction data with the proposed model was described; on the contrary, the message of the publication was the ‘untapped potential’ that should be realized if a method could be found to fit the simulation to experimental data.

The program package *nXDS* (Kabsch, 2014 ▸) is another software suite to process serial crystallographic data. The partiality model used assumes an isotropic Gaussian decay of the partiality with the angular offset from the ideal diffraction condition, making for simple symbolic expressions using Gaussians in 1D and a straightforward optimization of the parameters.

A different approach to computing the integrals that are required for estimating the partiality of reflections in still diffraction patterns uses ray-tracing principles (Kroon-Batenburg *et al.*, 2015 ▸). This approach is much closer to what would be called Monte Carlo integration outside of crystallography.

An isotropic and simplified partiality model using multi-dimensional but isotropic Gaussian basis functions has been implemented in *CrystFEL* and is the default for predicting spot locations and qualitative visibility since version 0.9.0. It uses a simplified version of equation (33) below, but without squaring the exponential term. The scalar projection of the covariance matrix orthogonal to the Ewald sphere is especially simple to calculate in this case. This model can also be used as a partiality model like xsphere and it is selected with the keyword ggpm. This model is most comparable with the one used in *nXDS* (Kabsch, 2014 ▸). Notable differences to that model are the formulation using the 3D Gaussian function and the concept of reciprocal peak width, which ascribes an additional constant width to peaks in reciprocal space independently of beam parameters and mosaicity, an effect that is especially significant at low resolution.

The Gaussian-like appearance of peaks on the detector possibly inspired Mendez *et al.* (2020) ▸ to impose a Gaussian decay of intensity with distance from the ideal diffraction condition on the detector. The result in equation (4) of Mendez *et al.* (2020) ▸ is seen to be proportional to a special case of equation (14) of this work when the covariance matrix 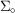 is uniform in all dimensions and scaled appropriately. Conversely, the result presented in this paper can be seen as a multi-dimensional generalization of the approach of Mendez *et al.* (2020) ▸. The significance of this difference becomes most obvious when considering elongated peak shapes in pink-beam experiments, which cannot be modelled by the approach of Mendez *et al.* (2020) ▸, owing to the isotropic nature of that model.

Dilanian *et al.* (2016) ▸ imposed a peak shape on the detector to fit the whole pattern in a similar manner to Mendez *et al.* (2020) ▸, but instead of an isotropic Gaussian shape they used an isotropic pseudo-Voigt shape. Pseudo-Voigt functions allow more heavy tailed shapes, and are thereby able to match shape transforms better with their asymptotically inverse-quadratic decay. However, their derivation does not connect these peak shapes with anything but the shape transform of the crystals. Our method generalizes a similar approach to non-isotropic peak shapes and connects them to mosaicity, non-monochromaticity, the crystal shape transform, the convergence and allows arbitrary compositions thereof. However, it is less general in the sense that only Gaussian shapes are employed. This is a deliberate limitation, because of the analytical difficulties that would be encountered with operations on anisotropic Cauchy distributions.

## 3. Derivation

### 3.1. Underlying diffraction theory

The incident wave interacts with a 3D object, which is described by its scattering potential, which in turn is mainly determined by its electron density ρ. In the Born approximation and a monochromatic incident wave with flux 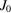 (in units of energy per area), the photon flux density *j* (in units of energy density as a function of solid angle) at each point on the detector can be described as the Fourier transform of the electron density 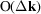 , evaluated at points corresponding to the difference 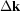 between the incident wavevector 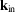 and scattering wavevector 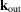 , a term *C* correcting for polarization effects (Cowley, 1995 ▸) and the scattering cross section as a proportionality constant.

The vectors 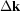 lie on a sphere with a radius ν reciprocal to the wavelength λ. This sphere is called the Ewald sphere and an equivalent result is known as the Fourier diffraction theorem (Slaney & Kak, 1985 ▸): 

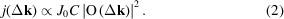

In this approximation diffraction is a linear operation, which means that the superposition principle applies to the complex wavefunction of the diffraction. The diffraction of several objects is the sum of the diffraction of these objects. The diffraction of an object by multiple sources is the sum of the diffraction of the object by each source. Depending on whether there is a fixed phase relation between the different contributions to the total diffraction, the contributions add incoherently (assuming an integration over a time interval several times the duration of the oscillation of the wave), that is as modulus squares, or coherently, which is in the complex domain, before the modulus square operation. For a derivation of the resulting average amplitudes of coherently and incoherently interacting waves see Section 1.3.2. of Cowley (1995) ▸.

### 3.2. Decomposition into Gaussian basis functions

Distributions of the sources and the objects are just an even further generalization of the superposition principle; combining these distributions amounts to convolutions of the distributions. However, 3D integrals of distributions over potentially curved paths do not, in general, have a closed solution. Numerical solutions are easy to determine, but compounded, derivative or derived properties (such as those required for least-squares minimization) grow in complexity, exponentially. Once one step is numerical, the next steps will most likely have to be numerical too. It is therefore useful and more insightful to have simple closed-form approximations. Gaussian distributions, as well as products and sums thereof, have closed and simple integrals when integrated over the whole domain or along a cut or a projection. Such integrals can likewise be expressed as a sum of Gaussian functions and a constant term. Also their Fourier transforms are well behaved. This way, integrating over multiple distributions still increases the complexity of the result, but starting from a less complex baseline. This means that if one can express all distributions in the model as a sum or series of Gaussian kernels, the conditional integration of the resulting distribution can be achieved symbolically. While not every distribution is suitably expressed as a weighted sum of Gaussian distributions, a large family is (Sorenson & Alspach, 1971 ▸). Many natural distributions belong to this family. And for most distributions used in the application of the method discussed here, the number of Gaussian basis functions, for sufficient approximation, is very low. The probability density function of a Gaussian distribution will be abbreviated with 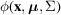 when convenient. All vectors are given in bold font and are column vectors unless transposed with a superscript T. The multiplication sign is omitted, except when equations are broken over more than one line, and multiplications between vectors or matrices are matrix multiplications by default.

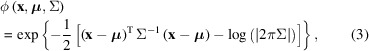

where ϕ is the probability density function of a Gaussian distribution, **x** is a point in space, 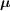 is the mean vector, Σ is the covariance matrix.

When the Gaussian basis functions are scaled appropriately, we refer to them as Gaussian kernels, as they are not normalized to one, like Gaussian distributions would be. This paper uses some common properties of probability distributions in general and Gaussian distributions in particular, which are summarized here. The joint probability of several uncorrelated outcomes is given by the product of their probabilities. By analogy, the probability density that satisfies all individual probability distributions is computed by a pointwise product of the individual densities. The product of Gaussian distributions is a scaled Gaussian with a mean given by the 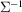 weighted arithmetic mean of the individual means and a new covariance given by the inverse of the sum of those weights: 

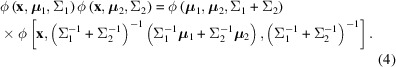

This result can be simplified further, when both densities are identical: 

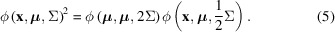

The probability distribution for the sum of two independent random variables is given by the convolution of the individual distributions. The rules for combining the means and variances are equivalent to the commonly employed error propagation: the means add, just like the variances. 

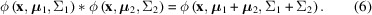

If the individual distributions are correlated, the means still add to form the sum, but there is an additional summand for the variance of the sum, 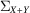 = 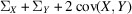 . In case of a correlation of 1 this reduces to 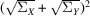 .

The identities of equations (4) and (5) can be used to compose the expected flux in a particular diffraction direction from the individual contributions of the source and of the object (see Fig. 1 ▸). As mentioned above, the formulation of this composition depends on whether the distributions are assumed to be in a fixed phase relation (coherent), or to have a randomly varying and uncorrelated phase shift (incoherent).

The following two identities, each first expressed using exponential functions and then in terms of ϕ, are at the core of the method for analytical integration used in this work. The first is the integral of the product of two Gaussian densities, which is then squared (for coherent integration): 

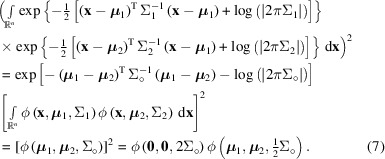

For incoherent integration the integration and squaring operations are reversed: 

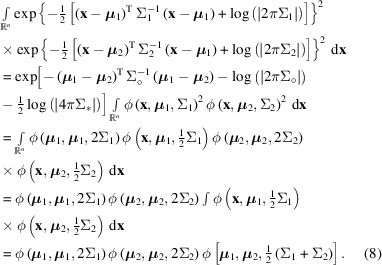

In equations (7) and (8) we have used the definitions: 

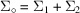




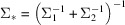




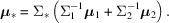

As can be seen from the above expressions, the difference between coherent and incoherent integration amounts to only a difference in scaling when both of the two distributions are single Gaussian distributions (that is, not sums of several Gaussians). As a simplification and because the linear scaling factor is hardly of any consequence, incoherent integration will be the default in the following, but the procedure can be applied with minor modifications for coherent integration as well. Partial coherence can be dealt with by splitting the coherent and the incoherent components into separate Gaussian functions and propagating them appropriately, or by interpolating between the coherent and the incoherent solutions based on the degree of coherence, but this will not be considered any further in this work.

### 3.3. Parametrization of the basis functions

#### 3.3.1. The illumination

**Figure 2:**
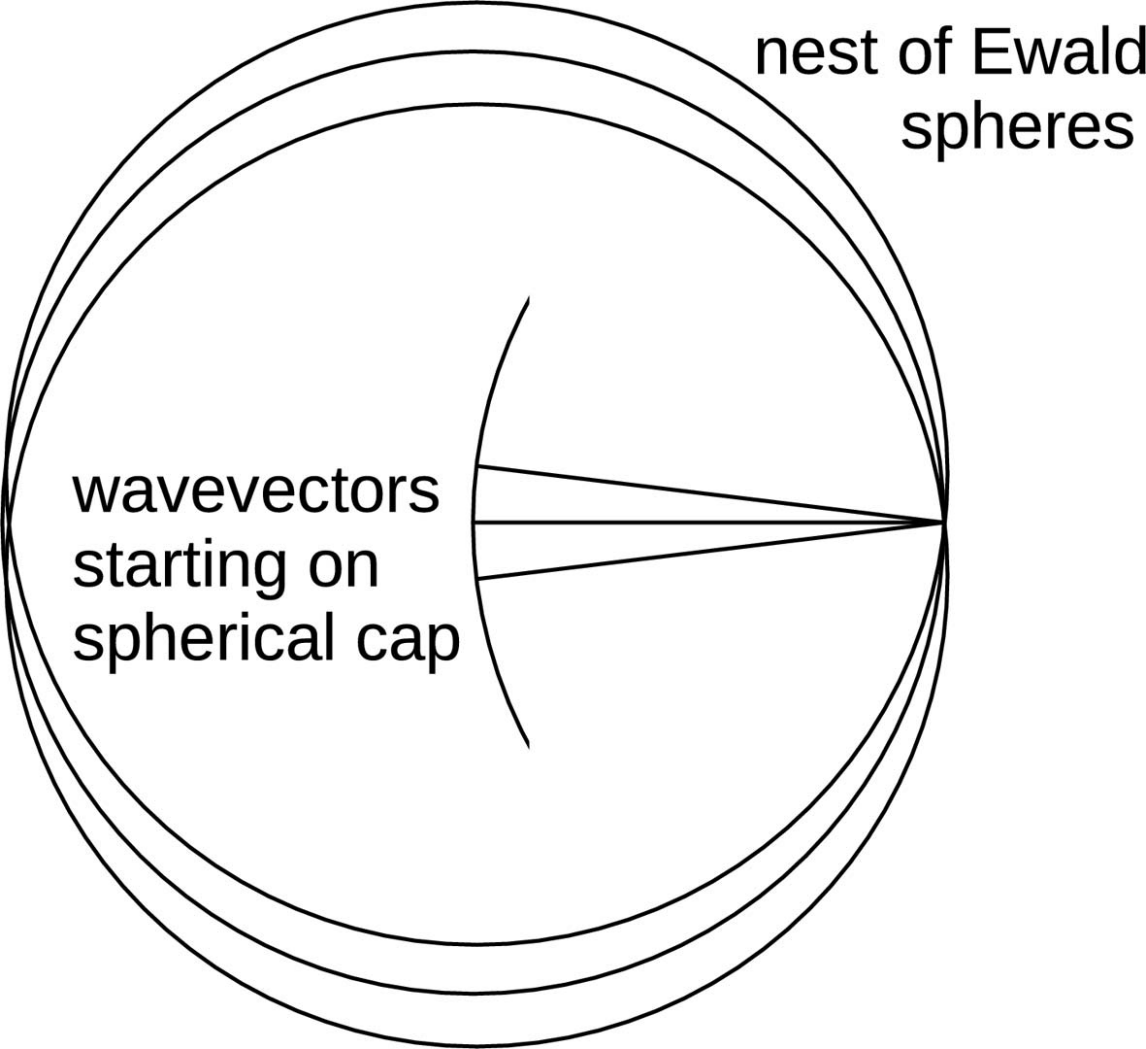
Illustration of the effect of divergence or convergence. Multiple (depicted three) incident-beam directions with the same wavelegth all lie on a spherical cap and produce a nest of Ewald spheres.

The diffraction condition, indicating the spatial frequencies of the object that contribute to the diffraction pattern and which is given by equation (2), forms a spherical shell that passes through the origin, which we have referred to above as the Ewald sphere. If the incident beam is convergent or divergent, there is a distribution of incoming directions, leading to a nest of spherical shells of equal radius in reciprocal space, whose centres lie on a spherical cap such that they all intersect at the origin. The normal at the centre of this cap is parallel to the mean beam direction (see Fig. 2 ▸). The covariance matrix 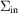 of 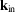 due to convergence or divergence alone cannot really be simplified in general, but if the distribution is isotropic, it can be written as

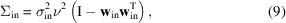

where 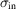 is the standard deviation of the incidence angles (*i.e.* the convergence), I is the identity matrix and vectors 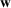 are unit vectors describing beam directions, derived from the wavevectors 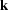 : 

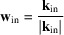




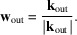

Each beam direction, in theory, would need its own polarization correction, and this could be achieved by integrating the polarization correction term for all the beam directions, but as small angles are assumed, the polarization correction of the main beam direction is deemed sufficient for all.

If there are multiple sources with different wavelengths, *i.e.* if the wavelength distribution has a finite bandwidth, the Ewald spheres have different radii and consequently the distribution of sphere centres, previously on a spherical cap, is broadened radially. The 3D distribution of sphere centres is approximated as a sum of Gaussian kernels. If the angular distribution is assumed to be small and independent of the distribution of wavelengths, it can be calculated by convolving the angle and wavelength distributions to form a cumulative distribution. The convolution of Gaussian kernels amounts to a summation of the respective covariance matrices [see equation (6)].

The distribution of 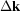 that samples the Fourier transform of the object in equation (2) and contributes to diffraction in a given direction, *i.e.* a point on the detector, can be derived from the distribution of sphere centres. The distribution of 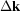 will be approximated as a Gaussian distribution with mean 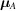 and covariance matrix 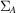 . Since the diffraction process does not change the wavelength, the outgoing wave distribution is perfectly correlated in wavelength with the corresponding incoming wave distribution. Differences of fully correlated Gaussian distributions require taking the difference of the square root of the respective covariance matrices. Given 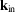 and 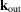 are approximated as Gaussian distributions, 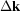 is distributed as a Gaussian around the mean value 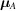 corresponding to the difference between the mean of 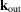 and 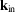 . The covariance matrix 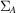 of the distribution of 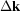 can be computed as the correlated difference between the distribution of 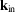 with covariance matrix 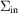 and the distribution of 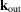 with covariance matrix 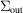 in that particular direction: 

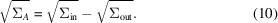

The distribution of 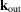 with the covariance matrix 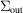 = 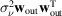 is not affected by divergence and only contains the wavelength distribution along 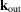 , and where 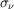 is the bandwidth. The distribution of 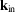 is affected by both the wavelength distribution and the angular distribution of incident beams, possibly correlated. In the slightly less general case, where it is assumed that the angular distribution of the incident beam is isotropic and is not correlated to its wavelength, the distribution of 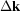 entirely due to polychromaticity is

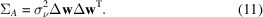

Combining this equation with equation (9) gives a way to estimate 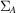 under simplified conditions: 

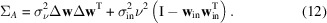

If we cannot assume that wavelength and incident angle are uncorrelated, 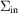 can be treated as a free parameter instead, and 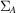 can be derived by rotating the component of 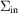 that is due to polychromaticity and therefore in line with the incident-beam direction to each 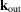 . The distribution of 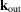 given 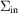 is therefore

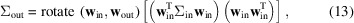

where rotate 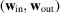 is the rotation matrix of the rotation around the axis orthogonal to 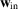 and 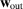 , that would align 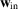 to 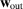 . Then 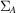 is given by equation (10).

#### 3.3.2. The crystal

Due to its periodicity, the Fourier transform of a crystal is concentrated in peaks. As discussed above, these peaks are broadened by properties of the crystal, such as the finite width of the crystal, mosaicity and strain. Here we define the separate effects that are modelled.

*Mosaicity* is commonly used to describe a rotational disorder of the crystal and can be seen as a distribution of orientations of the unit cell. Rotational disorder of an object in 3D will have six degrees of freedom in general: rotational disorder around three orthogonal axes and three covariance terms between them.

*Strain* is the distribution of contractions of unit cells. Generally, for each real-space lattice point in 3D, there can be a different distribution of displacements in the direction of the origin and transverse to it. In general, this is a 3D tensor. In the following we will assume that the changes of the structure factors due to strain are negligible.

Mosaicity and strain taken together, considering correlations of the effects in 3D, require a higher-dimensional tensor that maps each point of reciprocal space to a cross-correlation matrix. In the following, however, mosaicity and strain will be taken as uncorrelated and mosaicity will be assumed to be isotropic. This means that mosaicity is assumed to be equal in all angular directions and mutually independent of crystal strain. The integration in the following subsection (Section 3.4) will however be applicable with and without this simplification.

*Reciprocal peak shape* is the parameter that describes the distribution of each lattice point in reciprocal space, possibly due to the transform of the shape of a finite crystal, before being broadened by the effects of mosaicity and strain. In general this is a free parameter, but *e.g.* if the shape transform is 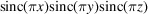 (the Fourier transform of a cube), it could be approximated by a Gaussian distribution with covariance 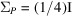 . Because of the approximately quadratic decay in the observed diffraction, as opposed to the exponential decay of the Gaussian, shape transforms are not approximated by sums of Gaussian functions efficiently. Therefore, if the reciprocal peak shape is the predominant effect that is broadening the diffraction condition, the approximate nature of the proposed model becomes most obvious. The strength of the proposed method is the ability to combine different effects analytically, where the convolved distributions naturally become smoother.

Integer multiples of the reciprocal unit-cell matrix *R* span the locations 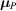 of the peaks in the Fourier transform of the crystal:

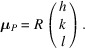

The density around 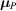 is approximated to be a Gaussian distribution with the covariance matrix 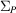 . The cumulative distribution results from the convolution of the individual distributions. Its covariance is therefore the sum of the covariance matrix 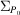 describing the shape transform, the effect of isotropic mosaicity 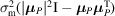 , and the effect of uncorrelated strain 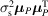 . Here 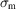 quantifies the mosaicity as the standard deviation of rotational disorder, and 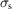 quantifies the strain as the standard deviation of the relative unit-cell size variation.

### 3.4. Evaluation of integrals

Given the distributions defined in Sections 3.3.1 and 3.3.2, we are now in a position to compute the diffracted flux density in a given direction 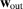 . This is done by evaluating particular integrals for each pair of Gaussian basis functions of the distributions, as given below.

Polarization and scaling terms were left out at this point for clarity, because they are not affected by the integration. If at least one of the distributions is assumed to have random or chaotic phases, the integration is incoherent, so using equation (8) and the definition of ϕ in equation (3) we get the following result: 

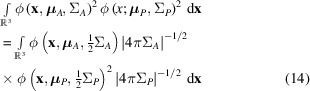




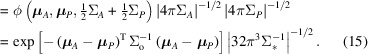

If all contributions to the diffraction described by the two distributions have a constant phase relation, the integration is coherent: 

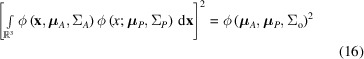




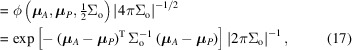

where 

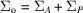




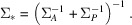




The result of equation (15) is applied below in Section 4 to compute a diffraction pattern that matches the observed pattern. This requires the appropriate scaling and polarization correction. All in all there are 17 parameters describing each Gaussian kernel of the crystal (nine for the unit cell, six for the shape transform and one each for mosaicity and strain) and nine describing each Gaussian kernel in the source (three parameters for the direction and six for a possibly correlated distribution of illumination angles and wavelengths). The source will typically not change for many crystals in a serial crystallography experiment and one Gaussian kernel will give enough degrees of freedom to describe the diffraction of each crystal.

## 4. Pixel-wise diffraction pattern prediction

The first way our approach can be used to process data is to model each pixel of a diffraction pattern, making use of as many constraints as possible in determining the hidden parameters and the structure-factor amplitudes. A still diffraction pattern can be calculated using the result of equation (15) for each point on the detector, by applying a polarization correction *C* and scaling with the intensity of the incoming beam and with the respective structure-factor modulus square 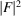 of each reflection: 

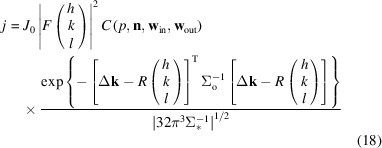




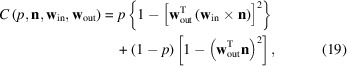

where 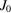 is the incident-beam flux, *p* the degree of polarization, **n** the normal to the polarization plane and *F* the structure factor. The flux measured in a pixel is the integral over all directions that fall into the solid angle of that pixel summed up for all Miller indices with significant excitation. If the predicted flux was constant over this area, the integral would be just proportional to the solid angle that the pixel occupies.

The detector is assumed to be composed of rigid panels. Each panel has its own 2D coordinate system consisting of the dimensions *fs* and *ss* defined in terms of the memory order, where *fs* (short for fast scan) is the dimension of values stored consecutively and *ss* (short for slow scan) is the dimension that is not. Each panel has a local coordinate system given by a 3 × 2 matrix *D* for the two dimensions in the plane of the panel and an offset vector **o** for the absolute position in space of the corner corresponding to the origin of the coordinate system of this panel. The solid angle of a pixel can be approximated using the derivative of the normed directionality vector 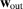 with respect to the detector coordinates: 

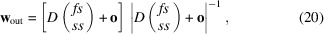

where 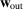 is the direction in which diffraction is to be predicted, *D* is the matrix translating between panel coordinates and spatial coordinates, **o** represents spatial coordinates of the reciprocal-space origin in detector coordinates, 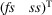 are the coordinates of the pixel on the detector.

For the following two derivations it will be useful to know the derivative of the direction 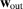 with respect to its two coordinates in the detector panel’s coordinate system: 

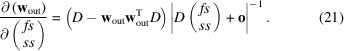

The solid angle Ω is approximated by the length of the cross product of the pixel sides projected onto the unit sphere: 

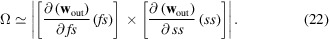

(Note that everywhere else besides in this equation the symbol × denotes a multiplication.) However, the predicted peaks can be very narrow, and therefore the predicted flux can vary substantially within a single pixel. To enable an efficient integration over the area, the predicted flux density can be smoothed analytically without changing the total flux of the whole diffraction pattern. This is achieved by introducing a Gaussian point spread function for the detector (the blue arrows in Fig. 1 ▸) with a covariance matrix corresponding to ½ the extent of a pixel, or for greater accuracy, by oversampling the pixel and applying the same procedure to the subpixels. Simply put, this smooths the prediction to a level where sampling it discretely only introduces minor artifacts, the main effect being a slightly reduced contrast. The constant ½, of the aforementioned pixel extent, minimizes the maximum Kullback–Leibler divergence 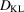 (Kullback & Leibler, 1951 ▸) between the desired proper integral (*b*) involving the error function and the estimate (*c*).

Equation (23) shows a proof in one dimension, that can be generalized to higher dimensions for all shapes for which an orthogonalizing coordinate transform can be found. It is natural to assume that the same constant approximately minimizes this difference even when the sides are not strictly parallel. The 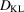 is an asymmetric measure for the difference of probability distributions taking into account that underestimating a probability is more detrimental than overestimating it. It was chosen because the predicted flux density is a scaled probability density. 

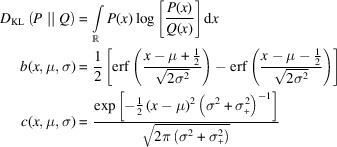




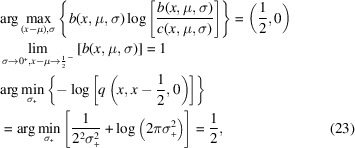

where *P* is the precise probability distribution, *Q* the approximation, μ the mean value, σ the standard deviation from the mean, σ_+_ the constant to be solved for. Using the results in equations (23) and (21) the resulting covariance matrix of the smoothing function is

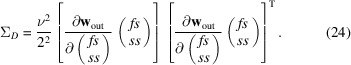

We now have a way of modelling the flux of each pixel. This is good enough for monochromatic experiments, but to model polychromatic experiments we need to take into account that detector response signals of integrating detectors are proportional to the total photon energy impinging on the detector. Integrating detectors are commonly chosen over counting detectors for SX (serial crystallography) experiments as they are not limited to measuring one photon per pixel at a time. The following derivation uses wavenumber ν, which is proportional to the impinging photon energy.

**Figure 3:**
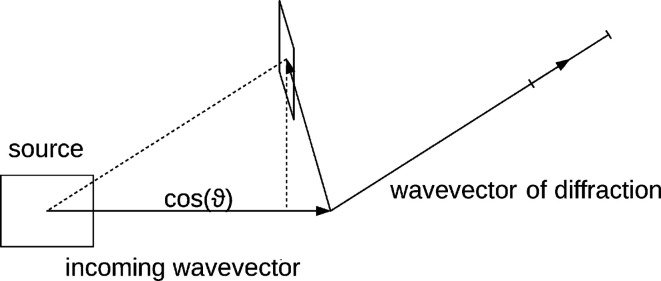
Geometric explanation for equation (25) for the expected wavenumber. Convergence, orthogonal to 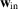 , and wavelength dispersion, in line with 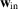 , are indicated as a box to highlight the shearing of the covariance when forming the correlated difference between 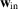 and 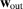 and their respective variances. It can be seen that the length of 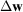 projected onto 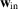 is 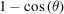 , where 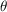 is the angle of diffraction.

The average wavenumber of the polychromatic diffracted beam at the particular location of a given pixel can be estimated from the mean point of the joint distribution of the source and the peak of the crystal in reciprocal space [compare equation (4)]. This is achieved by rescaling the component collinear to the incident beam. We are only interested in the collinear component because the deviation of 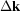 in any other direction is not due to the wavelength distribution but due to other factors like convergence. The rescaling is necessary, because the correlated difference between 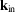 and 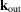 , which necessarily have equal wavelengths, leads to a covariance matrix of 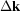 that appears sheared with respect to the covariance of 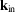 and compressed along the beam direction. A geometric visualization is offered with Fig. 3 ▸ in lieu of a mathematical proof. The cosine of the angle of diffraction equals the scalar product between the normalized incoming and outgoing wavevectors, leading to the following expression: 

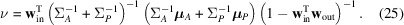




Having a distribution of photons of different wavelengths does not change the Poisson photon counting statistic, but it leads to an additional variance in the measured intensity proportional to the width of this distribution, because each photon measured can have a different energy. The width of the wavenumber distribution in each pixel can be estimated from the shape of the product of the two Gaussians in equation (14) by projecting to the incoming beam and rescaling. This is analogous to the expected wavenumber in equation (25). 

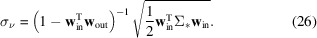

From the expected photon flux, the expected wavelength and the constant *g* describing the detector response as detector counts per wavenumber, the expected detector reading 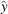 for a given pixel is given as the product 

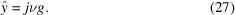

To model the photon counting statistic, whose variance scales with the expected photon count, and all degrees of systematic errors, of which the variance is assumed to scale quadratically with the predicted photon flux, we employ the following two-parameter (α and β) error model to predict the total variance: 

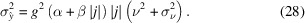

This error model is essentially equivalent to equation (3) of Diederichs (2010 ▸).

To connect the prediction 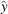 with the measured data 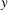 we introduce a probability distribution described by the density function 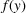 , which enables a maximum-likelihood optimization. The probability distribution is a mixed distribution of a smoothed Gaussian that approximates a discrete Gaussian with the additional variance 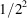 using the result in equation (23) and a super-heavy-tailed outlier distribution 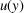 that models even extreme outliers like defective pixels: 

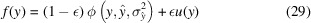




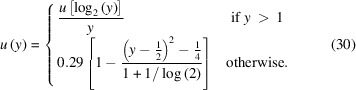




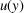
 is a smoother version of Rissanen’s universal prior for integers (Rissanen, 1983 ▸), ε is the outlier probability.

Crystal diffraction is sparse and most pixels will not see significant diffraction. The pixels with significant diffraction can be estimated conservatively by finding the potentially excited indices using a region growing algorithm (see Appendix *B* [App appb]) and then projecting the peak shape onto the detector [using equation (41)]. This accelerates the prediction greatly while not affecting the result in any significant way. Because derivatives can be computed analytically, the predicted diffraction pattern can be optimized using pseudo-Newton optimization methods like BFGS (Broyden, 1970 ▸; Fletcher, 1970 ▸; Goldfarb, 1970 ▸; Schanno, 1970 ▸) or gradient descent. In theory, this should make the optimization straightforward and efficient, but the target function has many local minima and plateaus.

**Figure 4:**
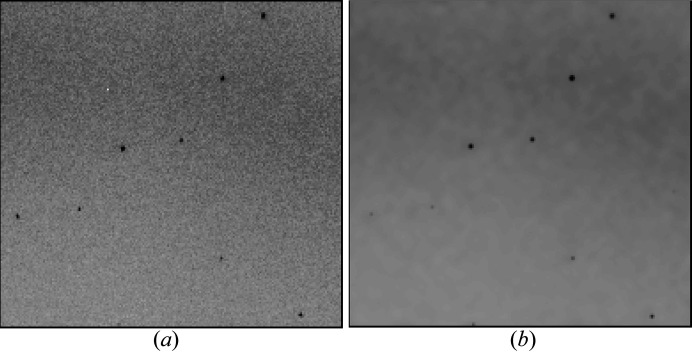
Comparison between (*a*) previously published diffraction data from a human serotonin receptor (Liu *et al.*, 2013 ▸) and (*b*) predicted diffraction of the same image region after successful optimization, with estimated background added. Diffraction is predicted using equation (19) with the substitution 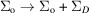 , corrected for the solid angle with equations (22), (25) to estimate the expected wavelength and summed up over all significantly excited Miller indices. Intensities are scaled according to the reference intensities deposited in the PDB (Protein Data Bank) under 4NC3 . The bandwidth of the X-ray beam is estimated to be about 0.1% [LCLS states 0.2% Δ*E*/*E* FWHM for the CXI beamline (LCLS, 2022 ▸)].

Together with the associated computational cost, this is the reason why pixel-wise refinement of the Gaussian sum model proposed in this paper so far has only been applied to individual diffraction patterns and not full data sets. This method also depends on a pixel-wise background estimate and a detector geometry that is determined well enough, such that predicted pixels coincide mostly with measured pixels. This demands the computation of about 8 kpx for a 4 Mpx detector. This makes it computationally expensive, requiring on the order of 10 single-core computing hours per pattern (4 GHz AMD A12). Therefore, this method has not yet connected with structure refinement directly, but is used to show visually that different diffraction patterns can be predicted accurately. Examples of successful pixel-wise diffraction pattern prediction after parameter optimization can be found in Figs. 4 ▸ and 5 ▸. Table 1 ▸ lists the parameters that were optimized.

## 5. Merging using integrated peak intensities

This section describes the second application of the model presented in Section 3: merge Gaussian partiality corrected integrated intensities (MGPCII). First we derive an expression for the total intensity of a reflection in a still diffraction pattern and then we describe a method of how to use this expression to reduce the detrimental impact of partially recorded reflections on the estimates of structure factors.

### 5.1. An expression for integated peak intensities

The total photon energy of one reflection can be computed by integrating the result of equation (15) over all directions. This integral can be approximated when considering that the angular extent of a reflection on the detector is small and the curvature as well as the change in width of the Ewald sphere is negligible for the integral over a single reflection. The density of the distribution of Ewald spheres can therefore be approximated as a planar (Winkler *et al.*, 1979 ▸) Gaussian, decaying along the direction of diffraction, but constant orthogonal to it. First the double integral is restated using equation (14). Then the integral along all possible outgoing wave directions is approximated with a projection onto the outgoing wave direction with the highest intensity 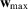 , which can be found by function optimization: 

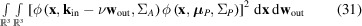




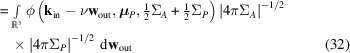




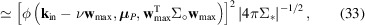

where 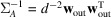 , *d* is the width of the Ewald sphere at the projection point. The photon flux of each reflection in each pattern is estimated as the product of the result of equation (33) with the incident photon flux 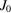 , the structure-factor amplitude squared, a linear scaling factor *a*, a *B*-factor correction term modelling a Gaussian decay of intensities due to random atomic displacements, and a term for the polarization correction [equation (19)]. This leads to an expression analogous to equation (18), but with an explicit linear and *B*-factor scaling instead of implicitly assigning those as terms in the structure factors: 

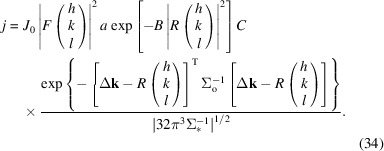

The calculation of the mean wavenumber is analogous to equation (25): 

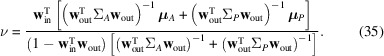

The width of the predicted wavenumber distribution is analogous to equation (26): 

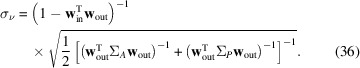

The expected detector count for each reflection is the product of wavenumber, flux and a detector constant, as in equation (27). Its variance is estimated with the same two-parameter error model as for the pixel-wise prediction in equation (28).

### 5.2. Parameter optimization for merging

The purpose of merging is to produce accurate estimates of the scattering intensities, proportional to the modulus squares of the structure factors, from a set of observed integrated peak intensities. To that end, to make use of equation (33), its free parameters need to be determined. The scattering intensities are among the parameters to be determined; the other parameters are listed in Table 2 ▸. To find the parameters we have chosen a maximum-likelihood approach because it can be more robust than least squares, but it is still relatively easy to optimize. The probability distribution to be optimized for each observation is 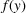 . Probabilities are assumed to follow a mixed distribution of a Gaussian distribution and an outlier distribution 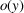 . The outlier distribution should be chosen so as to best describe all measured intensities in general, without prediction or scaling. In many cases, a Cauchy distribution is a good choice because it fits the shape of the distribution of integrated intensities well for frequently observed values and has an inverse quadratic decay like the positive intensities. The exact shape of the outlier distribution is less relevant; its most important feature is a slow asymptotic decay to make the maximum-likelihood approach robust. 

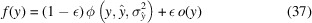




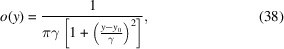

where *o* is outlier distribution, ε is outlier probability (1/16), γ is the scale parameter of the Cauchy distribution and 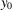 is 0.

### 5.3. Tests on experimental data

To show that equation (33) can be used to correct partially recorded reflections to improve the data quality, two serial femtosecond crystallography data sets were chosen. Data set 1 is a calibration data set of granulin microcrystals. This data set has not previously been published and was measured in October 2020 at the SPB beamline of the European XFEL in preparation for bacterial insecticide crystals, by a team led by Dominik Oberthür and Colin Berry. It has been deposited in the CXIDB with the ID 203. Data set 2 (Nass, 2020 ▸) allows SAD (single-wavelength anomalous diffraction) phasing.

**Figure 6:**
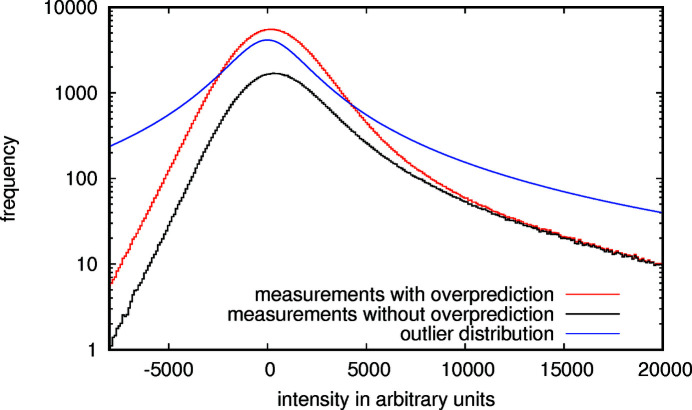
Histogram of measured integrated intensities of data set 1 in black (without overprediction) and red (with overprediction) overlaid with the Cauchy outlier distribution (γ = 1967.7) in blue. The outlier distribution was chosen so as to describe the measurements well, but also to reserve some probability especially for the extreme values. Note that the additional intensities due to overprediction are mostly small.

The diffraction patterns of both data sets were indexed and integrated using *indexamajig* of *CrystFEL* 0.9.1. To get a baseline for comparison with our method, the integrated intensities were merged with *partialator* 0.9.1 and *partialator* 0.8.0 using the partiality models ggpm, xsphere and unity, and the merged intensities were chosen that produced the best structure refinement results. The data sets were processed once with and once without overprediction, which is also integrating peaks further away from the diffraction condition, via the command-line option - -overpredict. The effect of overprediction is shown for the first data set in Fig. 6 ▸ and, as can be seen, the additional reflections are mostly of low intensity. Overprediction was not helpful when merging using *partialator* in any of the combinations of options that were tested. Therefore, overprediction is not enabled in the data points used as a comparison with the new method. However, it consistently led to better structure refinement results when correcting partialities using the generalized Gaussian diffraction model and maximum-likelihood parameter optimization during merging. This is why overprediction is enabled for that method.

The method described in Section 5.2 was applied to both data sets and the quality of the intensities was compared with the *partialator* baseline. In addition, data set 1 was investigated in more detail, with regards to overfitting, to the correlation of prediction and measurement and the distribution of estimated partialities, while the second data set was used to test how much SAD phasing could be improved.

**Figure 7:**
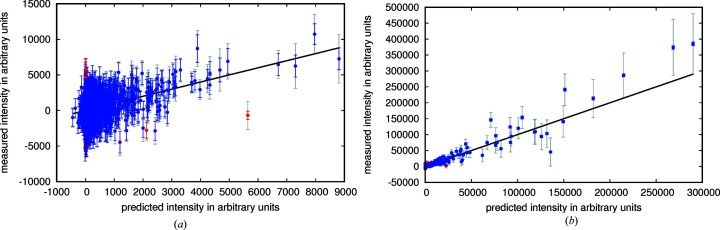
Predicted intensities versus measured intensities with the photon counting error estimates indicated by blue error bars and corrected error estimates by grey error bars. In red are data points that were treated as outliers, dots in blue were treated as regular data points. The black line shows where the points would lie if the predictions were in perfect agreement with the measurements. (*a*) shows the first 1000 intensities as recorded in the granulin data set (data set 1). (*b*) shows the intensities and predictions for the crystal with the strongest diffraction in the same data set.

After optimization of the scaling parameters (in Table 2 ▸) for data set 1, the correlation between prediction and measurement is high (Fig. 7 ▸), but the relative error between prediction and measurement still is about 25% and much larger than the photon counting error.

**Figure 8:**
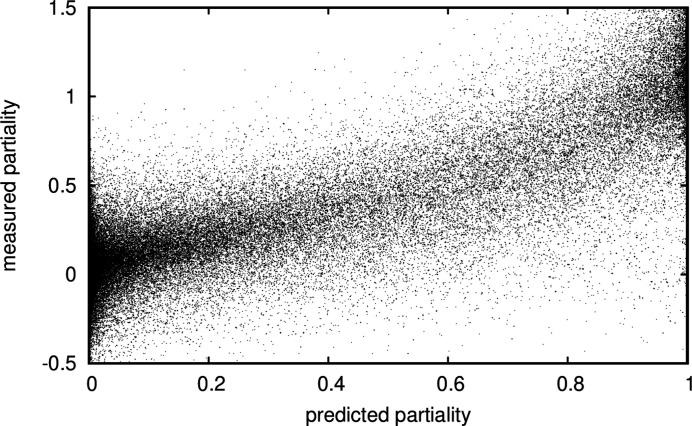
A scatter plot of a subset of predicted versus measured partialities with an estimated photon counting and background subtraction error of less than 1/8 in the granulin data set (data set 1). Chosen are the first 10 000 intensities from the data set in the order they are recorded, to make the result as reproducible as possible.

The comparison of predicted and measured partialities in Figs. 8 ▸ and 9 ▸ shows a strong correlation, which is exploited when correcting the measurements using the partiality estimate. Unknown partialities increase the variance of the intensities before merging and therefore of the merged intensities too. The variance can be reduced by partiality correction.

**Figure 10:**
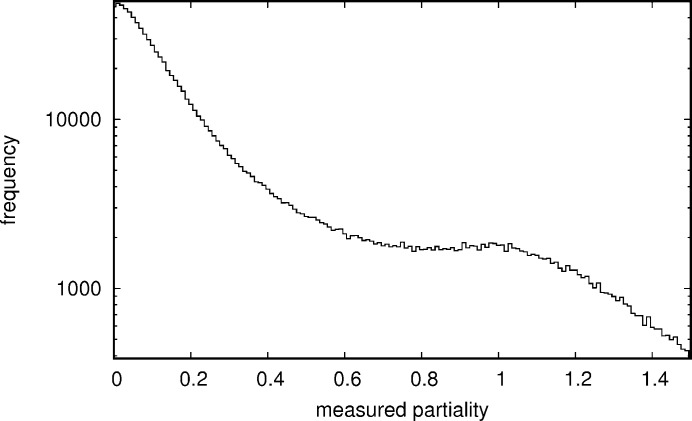
Histogram of partialities measured with an estimated photon counting and background subtraction error of less than 1/8 from the granulin data set (data set 1).

As would be expected for the smoothed distribution of the function values of a Gaussian function with uniform input (for a derivation of the distribution before smoothing see Appendix *D* [App appd]), the histogram of the measured partialities (Fig. 10 ▸) has an optimum at 0, corresponding to a reflection that was not observable (most reflections in a given crystal orientation are not observable), and also a very faint optimum at 1. The optimum at 1 corresponds to the flat top of the intensity curve of an observation near its maximum intensity.

**Figure 11:**
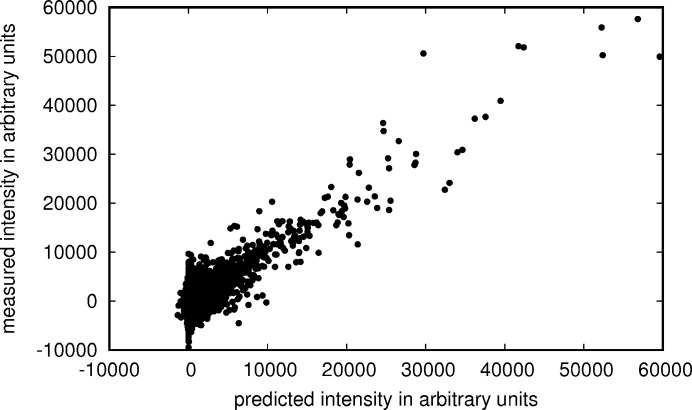
10 000 random pairs of predicted and measured intensities from the random half data set of data set 1 that was used to to fit all parameters.

**Figure 12:**
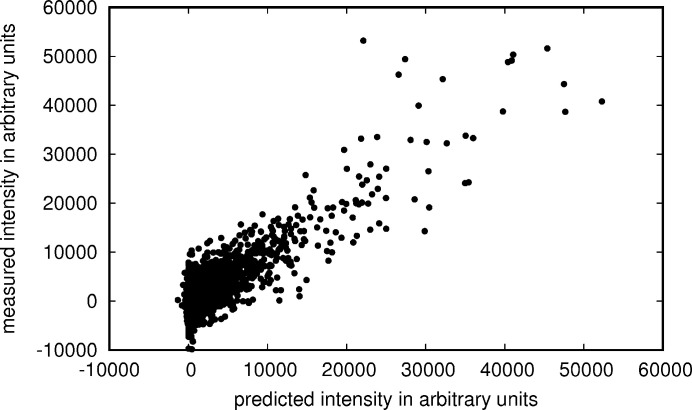
10 000 random pairs of predicted and measured intensities using the parameters determined from the random half data set of data set 1 used in Fig. 11 ▸. Note the slightly reduced correlation compared with Fig. 11 ▸.

**Figure 13:**
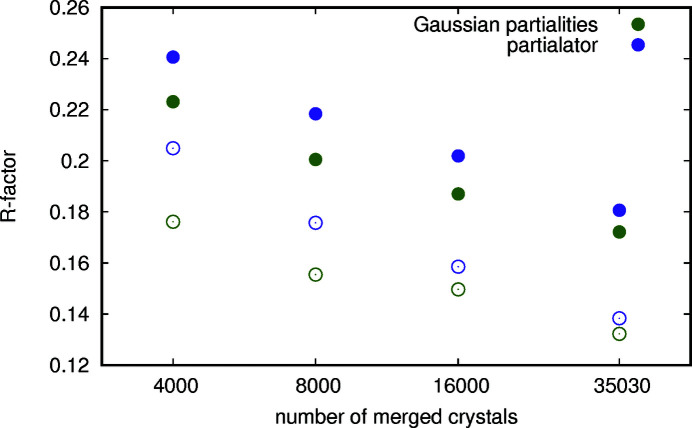
Comparison of structure refinement results of the granulin data set (data set 1) using *phenix* 1.18-3855 to a resolution of 1.8 Å of MGPCII, in green, and *partialator* 0.9, in violet. The bold dots represent the free *R* factor, the small circles represent the *R*_work_. The partiality model ggpm gave the best result for *partialator* for all sizes of subsets that were tested.

To test the amount of overfitting, data set 1 was split randomly in two halves. The first half was used to optimize the parameters of the scaling and partiality model in Table 2 ▸ and the second half was used to test the correspondence of prediction and measurement. The median correlation of 256 random prediction–measurement pairs (to increase the robustness of the correlation, as there are outliers that skew the correlation to 0, −1 or 1 randomly) decreased from 0.59 to 0.56; the reduction in correlation can be observed by comparing Fig. 11 ▸ with Fig. 12 ▸. This is evidence of some degree of overfitting, but also means that even half the number of peaks is sufficient to arrive at roughly the same prediction. So even though the method of partiality correction of integrated intensities would likely profit from additional constraints (among the constraints that were left unused are the peak positions on the detector and the fact that the different unit-cell matrices are mainly just different rotations of each other), it still reduced the number of diffraction patterns necessary to achieve a given data quality by about a factor of 2. *R* factors after automatic refinement (Fig. 13 ▸) were consistently lower for MGPCII than for *partialator*.

Data set 2 is of the adenosine receptor A_2A_, measured at LCLS (Linac Coherent Light Source) using a wavelength of 2.7 Å (Nass, 2020 ▸). The protein contains 22 sulphur atoms and the wavelength is close enough to the absorption edge to make SAD phasing possible. This makes this data set suitable to see to what extent partiality correction would improve phasing success. For all merged intensity files a SAD phasing attempt was run using *phenix.autosol* (Liebschner *et al.*, 2019 ▸) and the known protein sequence and a resolution cutoff of 2.3 Å.

**Figure 14:**
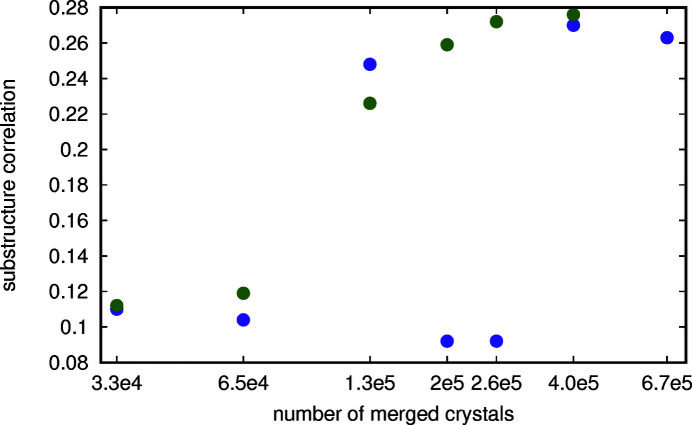
Maximum HySS correlation coefficient found during automatic SAD phasing using *phenix.autosol* from A_2A_ crystals (Nass, 2020 ▸) as a function of the number of crystals used during merging. The entries in green are for MGPCII, whereas the violet dots represent the results of *partialator*.

**Figure 15:**
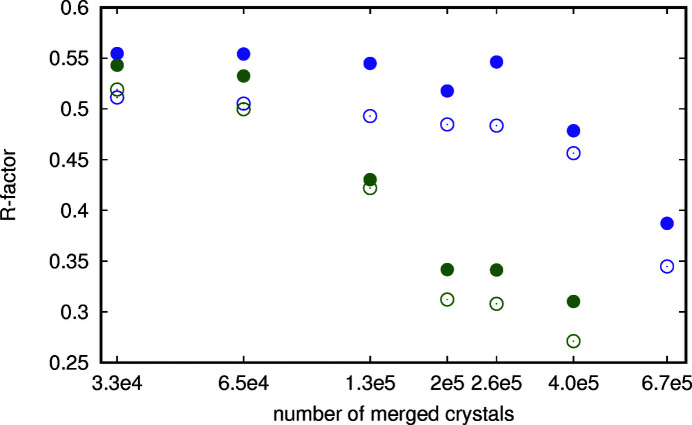
*R* factors of the refinement of structures built during automatic SAD phasing using *phenix.autosol* from A_2A_ crystals (Nass, 2020 ▸) as a function of the number of crystals used. The entries in green are for MGPCII, whereas the violet dots represent the results of *partialator*. The solid dots are *R*_free_ and the open circles are *R*_work_.

As can be seen from the hybrid substructure search (HySS) correlation coefficient in Fig. 14 ▸ and the *R* factors that the automatic structure building and refinement achieved (Fig. 15 ▸), the improved merging efficiency is reproduced for the anomalous signal too.

## 6. Discussion and conclusion

Using Gaussian basis functions, approximations were developed that have enough degrees of freedom to describe most of the significant effects in macromolecular crystallographic experiments. These approximations were used to simulate diffraction patterns, which were visually very similar to measured diffraction patterns. Partiality estimation and post-refinement using these functions have reduced the number of measurements necessary for a given data quality in merged intensities. In the first example it reduced the number of patterns required to achieve a given *R* factor by about a factor of 2 compared with *CrystFEL*’s *partialator*. In the second example S-SAD phasing succeeded with about a quarter of the diffraction patterns. The range of data sets that were tested is not comprehensive, however, and *partialator* is not the only alternative, nor necessarily the best program, just the most commonly used.

There are many differences between our method and *partialator*, partiality estimation being only one of them. Without exhaustive testing we are not able to tell precisely which differences provide the greatest improvement. A significant improvement can however be attributed to the error model used, which has been shown to improve merging on its own using a different approach (Brewster *et al.*, 2019 ▸). Another important difference is that our method profits strongly from overprediction, adding many measurements with mostly insignificant intensities, by integrating reflections even if they are further removed from the ideal diffraction condition. It may seem that overprediction should not improve the precision of the merged result as strongly as it does, especially when the added intensities are mostly small or negative. However, we find that the small intensity values outside the diffraction condition act as a powerful constraint for determining reciprocal peak shape, mosaicity and strain.

Even though polychromatic diffraction of mosaic crystals can be described qualitatively, automatic refinement has proven to be difficult so far because predicted peak positions can vary by more than half the inter-Bragg distances. There are many more applications for approximating diffraction with Gaussian basis functions in the way we described that remain to be explored. Pixel-wise refinements, as done in the program *diffBragg* (Mendez *et al.*, 2020 ▸), should lead to even better merging efficiency and a more precise detector geometry refinement at the cost of more computation time. The model could also be used to predict the intensity of peaks per frame in a rotation series and therefore simplify the visual examination of the effectiveness of data processing, especially for peaks lying along the axis of rotation.

Integrated peak intensities are less demanding for numerical optimization than pixel-wise intensities because there are many pixels per reflection. Furthermore, because peak intensities are integrated over a larger pixel area on the detector, the geometry description only needs to be accurate enough for most of the peak intensity to fall within the integration area. A consequence of integration is the drastic reduction of the number of constraints. Whereas pixel-wise optimization uses thousands of pixels, albeit with somewhat degenerate information (in a single Gaussian approximation each peak on the detector can be described with six variables: height, *x* and *y* coordinates of the centre, major and minor axis and orientation of the elliptical shape; oversampling the shape does not add constraints in this approximation), the number of constraints in a traditional cell parameter and orientation refinement during merging of serial crystallographic data sets is just high enough to be clearly overdefined. This might mean that for data sets of very weakly diffracting crystals and without additional constraints a pixel-wise refinement is the only option.

Lastly we want to emphasize the generality of this model. The same model can be used to simulate diffraction patterns and integrated intensities of serial monochromatic and polychromatic crystallography experiments. The analytical nature of this model makes analytical derivatives available, which is useful for mathematical optimization. It also makes deriving properties like peak locations and shapes and integrals over angular ranges and areas practical. Together this opens up a wide range of experiments where this model can be applied.

## Figures and Tables

**Figure 5:**
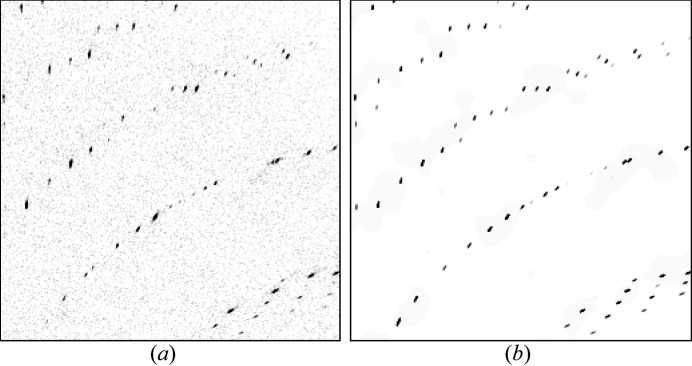
Comparison between (*a*) diffraction data (unpublished) of selenobiotine-bound streptavidin crystals and (*b*) predicted diffraction of the same image region with estimated background added. Diffraction is predicted using equation (19) with the substitution 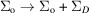 , corrected for the solid angle with equations (22), (25) to estimate the expected wavelength and summed up over all significantly excited Miller indices. The diffraction was measured at ESRF with a 1M Jungfrau detector using a pink beam with 5% bandwidth FWHM. The structure factors for the prediction are taken from the streptavidin–norbiotin complex structure deposited under 1LCV in the PDB (Pazy *et al.*, 2002 ▸).

**Figure 9:**
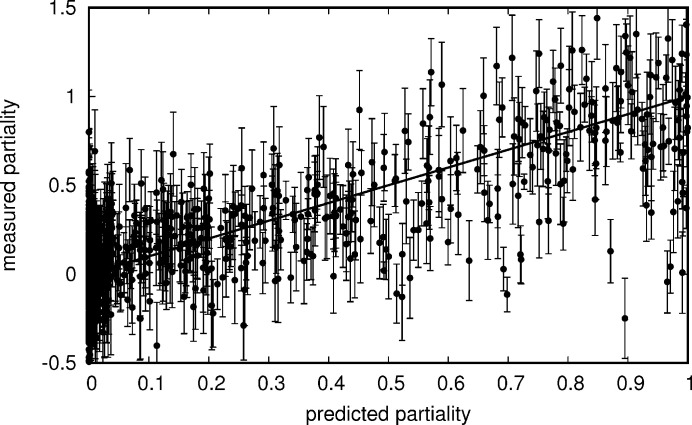
Predicted partialities compared with measured partialities, with photon counting error estimates indicated by error bars. The first 993 values from data set 1 in the order they are recorderd to have an estimated photon counting and background subtraction error of less than 1/4 are displayed. The black line shows where the points would lie, if the predictions were in perfect agreement with the measurements.

**Figure 16:**
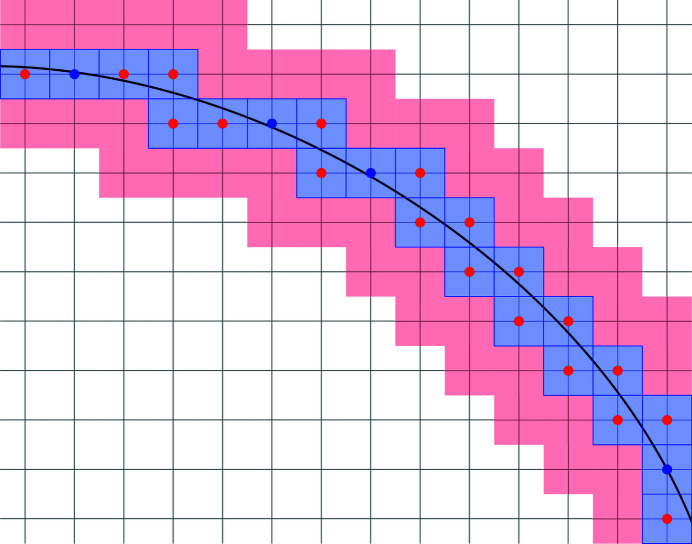
An illustration of region growing for identifying reflections with significant contribution to the diffraction. The grey gridlines intersect at integer combinations that are the Miller indices of the reflections in reciprocal space. The Ewald sphere, or diffraction condition more generally, is assumed to be a smooth function and much thinner in one dimension than the others. It is caricaturized with an ellipse sector in black. The algorithm starts at any of the light red or light blue squares. For each blue square that intersects with the diffraction condition at any point, the diffraction condition at the exact Miller index is evaluated. A significant contribution is indicated with a blue dot, an insignificant contribution with a red dot. For each blue square all new neighbours are inspected for intersections in the same manner. Squares that do not intersect the diffraction condition at any point are coloured in light red and do not prompt the inspection of their neighbours.

**Table 1:** Parameters that were optimized against pixel values for each image

Parameter	Degrees of freedom	Optimization
Geometry description	9 for each panel	Yes
Unit cell	9	Yes
Reciprocal peak shape	6	Yes
Mosaicity	1	Yes
Strain	1	Yes
Linear scale factor	1	Yes
*B* factor	1	Yes
Error model	2	Yes
Source description	10	No

**Table 2:** Parameters that were optimized against integrated intensities for each crystal

Parameter	Degrees of freedom	Optimization
Geometry description	9 for each panel	No
Unit cell	9	Yes
Reciprocal peak shape	6	Yes
Mosaicity	1	Yes
Strain	1	Yes
Linear scale factor	1	Yes
*B* factor	1	Yes
Error model	2	Yes
Source description	10	No

## Appendix A Derivatives and derived properties

### A1. Peak shape on the detector

Looking at the predicted intensity as a function of the position  on the detector, and assuming that the peak intensity falls into a small angular range (<10°) where the covariance matrices can be approximated as locally constant with good accuracy, an approximation of the peak shape on the detector can be derived by factoring out the (approximately) constant terms from the exponential. For straightforward computation and best approximation, the direction with the highest intensity  should be determined; this can be achieved with any function optimization algorithm. Newton’s method is equivalent to iteratively completing the square for the exponential term and, because the target function can be made very nearly quadratic by taking the logarithm, it converges very quickly. The detector coordinate system is commonly given by a 2-by-3 transformation matrix *D* and an offset vector **o**. The outgoing wave direction is therefore given by the normed position vector:

The point  denotes the peak position on the detector, *i.e. *the position of maximum flux. Using  the normed directionality vector can be approximated to first order as

Equation (14) for the flux on the detector can be expressed as a scaled Gaussian (or a sum thereof), and using the linearized expression for the directionality vector the intensity on the detector can be expressed as

with *c* the proportionality constant.

The scaled Gaussian, which only appears to be 3D, can be rearranged to show the 2D form using suitable substitutions:

if the outgoing wave vector was optimal,

The peak on the detector can therefore be approximated by a scaled 2D Gaussian (or several), potentially broadened by the point spread function of the detector. The shape (without broadening) is given by the 2D covariance matrix  .

## Appendix B Asymptotically optimal prediction of a diffraction image in areas with flux above threshold

The naive approach to calculating a diffraction image of a snapshot would be to compute the multiplication of the source with the object and the convolution with the Green’s function via the FFT (fast Fourier transform). This holds in general in kinetic far-field approximation even for non-crystals. For a crystal the Fourier transform is sparse and this is usually exploited by iterating over the Miller indices. The computational complexity is  where *N* is the number of indices to be considered in each direction. Because the Ewald sphere essentially is 2D we can come up with a solution to compute this in  by using a region growing approach. Every reflection that exceeds a threshold has at least one neighbouring Miller index which has a virtual reflection at most as far as half the inter-Bragg distance that would exceed the threshold. Fig. 16 ▸ pictures a curve with some width going through a mesh. The curve intersects only some nodes of the mesh, but for every node that it does there is at least one face (or enclosed volume for higher dimensions) that it intersects. The path of the curve can be traced by testing neighbouring faces (or volumes) for intersection iteratively.[Chem scheme1]

To implement the set operations efficiently and to actually achieve  asymptotic complexity, the hash table patchmap (Brehm, 2019 ▸) was used, but most other data structures with amortized constant lookups and insertions would do as well because the limiting step is checking the overlap in step 4.

### b1. Maximum flux of a virtual reflection in the range (*h*±½, *k*±½, *l*±½)

The maximum flux of any virtual reflection with fractional coordinates closer to a given Miller index  than any other Miller index can be conservatively estimated by taking the reflection at  , and convolving its location with a width equal to one unit of  in reciprocal space while not changing the normalization of equation (33). This distance corresponds to a covariance matrix equal to half the reciprocal unit cell times half the reciprocal unit cell transposed – note the similarity to the result in equation (23). The approximation is not sensitive to the assumed direction of maximum diffraction intensity  , a rough estimate is sufficient. For compactness the term  will be combined as  :

### b2. One frame in a rotation series

The integrated intensity in a given outgoing direction  can be expressed as proportional to a Gaussian function [see equation (8)]. The intensity integrated over an oscillation range  is then proportional to

where  is the rotation matrix with angle α, *U* is the unit-cell matrix (real space). It can be evaluated by first finding the index values that will be excited to a significant degree in the outgoing arc section described by the position on the detector, the axis of rotation  and the oscillation range. Then the target function can be approximated by a 1D Gaussian by developing a small-angle approximation around the rotation with the highest predicted intensity and factoring out the constant terms of the 3D Gaussian. This 1D Gaussian integrated for the given range yields a difference between two error functions:  is the rotation matrix that yields maximal diffraction,

A similar result is stated with equation (37) in section 3.6 of Kabsch (2014 ▸).

## Appendix C Pixel-wise backgound estimation

Background estimation for the pixel-wise diffraction prediction was done by minimizing the following function that acts similarly to a boxed median filter or a boxed mean of the middle 75%, which are both much easier to compute, but less flexible and slightly less smooth:

α was typically 1/4, *u* is the outlier distribution from equation (30) and  are the pixel values. The function is minimized by finding the optimal values for  and  . The indices enumerate the pixels and the second sum goes over all pairs of adjacent pixels. This approach is very likely overcomplicated, but it did not turn out to be a bottleneck and was good enough.

## Appendix D Theoretical distribution of partialities

If the intensities of reflections decline like a Gaussian function when leaving the optimal diffraction condition, and because the diffraction condition is essentially random, the distribution of partialities should look like the distribution of function values of a Gaussian distribution with uniform input. The Gaussian function, scaled to a peak height and variance of 1, is  . Its inverse function, not to be confused with the inverse Gaussian distribution, is  =  . There is an ambiguity because  is not strictly increasing or decreasing, but it is symmetric around the axis *X* = 0, and we can therefore restrict the analysis to the increasing branch only. The random variable *X* is assumed to be uniformly distributed on some region symmetric to 0 on a support  . The probability density therefore is  and the cumulative distribution function  . The cumulative distribution function of the random variable  is the distribution function of *X* applied to the inverse function of *g*:  =  =  , which is  . The density function is its derivative,  . In the limit of a large interval  this is not a proper density function any more, as the integral  is divergent.
